# Angiogenic potential of clonal populations of human mesenchymal stem cells

**DOI:** 10.1186/1753-6561-6-S4-O19

**Published:** 2012-07-09

**Authors:** J Scott, A Liew, G Shaw, M Murphy, F Barry, T O'Brien

**Affiliations:** 1 School of Medicine, College of Medicine and Health Sciences, National University of Ireland, Galway, Ireland

## Introduction

Critical Limb Ischemia (CLI) is the most severe form of peripheral arterial disease. It is a highly prevalent condition with suboptimal therapeutic options. One third of CLI patients are not suitable for surgical revascularization - these 'no-option' patients have substantial morbidity and mortality and frequently have diabetes mellitus. Hence, new treatments are urgently required. Mesenchymal Stem Cells (MSCs) are a novel and exciting potential for inducing therapeutic angiogenesis. However, MSCs are heterogeneous in nature and the angiogenic potential of different clonal populations from the same donor is currently unknown. We hypothesize that MSC heterogeneity will allow for the isolation of clonal cell populations with enhanced angiogenic potential. These populations may have enhanced therapeutic potential and may also be of interest in exploring the mechanism of MSC-induced angiogenesis.

## Methods

The angiogenic potential of three daughter clones and the parent clone from a single donor were determined. Firstly, in vitro angiogenesis assay was performed. Next, the endothelial differentiation ability of these clones was assessed. Finally, the nature of angiogenic products in the secretome was assessed by performing an antibody array analysis on the conditioned media collected from each clone.

## Results

Daughter clone 1 and 2, as compared to the parent and daughter clone 3, were highly angiogenic demonstrating the existence of clonal heterogeneity. We next went on to examine the ability of these clones to undergo endothelial differentiation and observed that these two clones also showed significant endothelial differentiation capability which was greater with clone 1. Clone 1 which has the greatest angiogenic and endothelial differentiation capacity secreted the greatest quantity of angiogenic factors.

## Conclusions

MSC clones derived from a single human donor are highly angiogenic but individual clones vary in their angiogenic potential. This study suggests that therapeutic efficacy of MSC populations in ischemic states may be enhanced by the use of angiogenic clones.

